# Investigating Head and Neck Cancer Survivors’ Experience of Survivorship Care

**DOI:** 10.1093/geroni/igaa057.1391

**Published:** 2020-12-16

**Authors:** Aaron Seaman, Seyedehtanaz Saeidzadeh, Nicholas Kendell, Alan Christensen, Timothy Thomsen, Heather Reisinger, Nitin Pagedar

**Affiliations:** 1 Carver College of Medicine, University of Iowa, Iowa City, Iowa, United States; 2 College of Nursing, University of Iowa, Iowa City, Iowa, United States; 3 East Carolina University, Greenville, North Carolina, United States

## Abstract

Head and neck cancer (HNC) accounts for 4% of all cancers diagnosed in the US, with 75% in adults over 55 years of age. HNC survivors must deal with the long-term consequences of the cancer and its treatments, which can have significant long-term physical, psychosocial, and financial consequences, dramatically impacting survivors’ lives. While research has identified the unmet needs of HNC survivors, there has been little examination of HNC survivors’ experiences living with a cancer history and engaging in survivorship care. To explore survivors’ experiences and their attitudes toward their survivorship care, we conducted in-depth, semi-structured interviews with 22 HNC survivors whose survivorship care was managed within the HNC program of an academic tertiary care institution. Participants’ mean age was 65 years old, ranging from 33 to 86. The majority of the participants were male (68%), white (96%), married (81%), and had some college education or a higher degree (81%). One third of participants (n=7) had rural residence, as defined by the Rural-Urban Continuum Codes. Participants reported a wide range of experiences based on multiple factors: cancer site, staging, and treatment; their expectations prior to treatment; and personal and social context. They varied in their approaches toward understanding and incorporating the impacts of their cancer experience, from physical side effects of treatment to social impacts. They described the importance of survivorship care both in physical and social terms. We discuss the implications of these results for future interventions to improve HNC survivorship care delivery.

